# PHIPing along: Evolution of PHIP as a cancer biomarker and a target for therapy

**DOI:** 10.18632/oncotarget.26341

**Published:** 2018-11-16

**Authors:** Mohammed Kashani-Sabet, Vladimir Bezrookove, David de Semir

**Affiliations:** Center for Melanoma Research and Treatment, California Pacific Medical Center (CPMC) Research Institute, San Francisco, CA, USA

**Keywords:** melanoma, PHIP, cancer biomarker, targeted therapy

Recent studies have assigned novel roles to pleckstrin homology domain-interacting protein (PHIP) in tumor progression [1, 2]. PHIP was initially shown to operate in the insulin-like growth factor receptor 1 (IGF1R)/PI3K signaling pathway in normal pancreatic islet cells [3]. Gene expression profiling identified *PHIP* as the top gene overexpressed in metastatic *versus* primary melanomas [4], prompting us to better comprehend its role in melanoma and other cancers.

Initial studies showed PHIP to be both a marker and mediator of melanoma distant metastasis [5]. Immunohistochemical (IHC) analysis of PHIP expression showed an independent role in predicting survival associated with primary melanoma. *PHIP* silencing resulted in significantly decreased melanoma cell growth and invasion, with a concomitant suppression of the metastatic potential of melanoma cells. The survival of mice injected intravenously with melanoma cells expressing anti-*PHIP* shRNA was prolonged, establishing the rationale for therapeutic targeting of PHIP. *PHIP* copy number was elevated in a subset of melanomas using fluorescence *in situ* hybridization (FISH) analysis. This was somewhat surprising, given that the *PHIP* gene is located on 6q14, with earlier studies reporting losses of 6q in melanoma [6].

**Figure 1:**
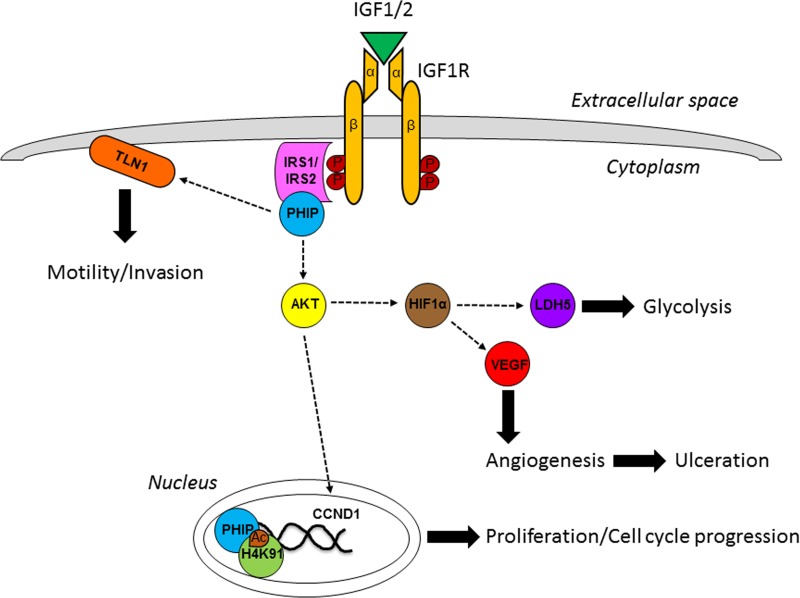
Involvement of PHIP in the IGF1R pathway

Subsequently, we assessed the prognostic impact of *PHIP* copy number [7]. Elevated *PHIP* copy number significantly and independently predicted survival in primary melanoma, confirming the results obtained using IHC analysis. In addition, there was a significant correlation with ulceration, a powerful histologic prognostic factor incorporated into the American Joint Committee on Cancer (AJCC) melanoma staging classification. While the prognostic importance of ulceration is well appreciated, the biological basis for its significance in melanoma progression is poorly understood. Functional studies in human melanoma cells showed that PHIP promotes glycolysis and angiogenesis [7] by regulating HIF1A, LDH-5 and VEGF (Figure 1). Intriguingly, as serum LDH levels are also incorporated into the AJCC staging classification, our studies indicated that activation of the same signaling pathway could account for the biological basis of two key prognostic factors for melanoma.

In a recent study [1], we undertook a comprehensive analysis of the biomarker role of *PHIP* copy number in melanoma. We developed three distinct tissue sets: cohort 1- 204 primary melanomas; cohort 2- 130 node-positive melanomas; and cohort 3- a matched set of 15 patients with primary and metastatic melanomas from patients who experienced both lymph node and distant metastasis. In cohort 1, we confirmed the independent prognostic impact of *PHIP* copy number in a cohort of melanoma patients derived from a separate population, and validated its significant association with ulceration.

In cohort 2, we identified the molecular subtypes of melanoma with elevated *PHIP* copy number. *PHIP* copy number was significantly increased separately in intact PTEN-expressing, in *NRAS*-mutant, and in *BRAF*-mutant coupled with intact PTEN-expressing melanomas. The enrichment of PHIP in PTEN-expressing melanomas was understandable, since both proteins operate in the PI3K pathway, suggesting that either molecular aberration is sufficient to result in pathway activation. However, PHIP enrichment in *NRAS*-mutant melanomas was surprising, as *NRAS* mutations are thought to activate both the MAPK and PI3K pathways, thereby suggesting functions for PHIP outside of the PI3K pathway. The enrichment of PHIP in *BRAF*-mutant, PTEN-expressing melanomas was also important, as few other oncogenic drivers have been described in this well-characterized melanoma subtype.

Our studies in cohort 3 assessed whether *PHIP* copy number elevations were present in the transition from primary melanoma to lymph node and distant metastasis. Remarkably, in the cases in which triple-matched specimens were available, there was a monotonic increase in elevated *PHIP* copy number, such that the mean copy number (i.e., percentage of melanoma cells harboring 3 or more copies of *PHIP*) increased from 13.5% (in primary tumors) to 21.6% (in lymph node metastases) to 55.5% (in distant metastases). Key aspects of these analyses were confirmed by analyzing The Cancer Genome Atlas (TCGA) cohort.

Our data support the notion that PHIP is clonally selected in the transition from primary to metastatic melanoma. These copy number variations are caused by chromosome instability, which is both a hallmark of cancer and associated with increased metastatic potential [8]. The clonal selection of *PHIP* identifies it as a biomarker on many levels, as it can be used to classify molecular melanoma subtypes, or to define disease stage or severity.

In a separate recent study [2], we analyzed the role of PHIP in three cancers (breast and lung cancer, and melanoma) in which targeted therapies have shown the greatest efficacy. We focused on molecular subtypes of these cancers (termed “driver-negative”) lacking the known molecular drivers or targets for therapy, which are largely different in the three tumor types. We showed that PHIP promotes the progression of driver-negative subtypes of these three cancers, in part by activating AKT, TLN1, and CCND1 (Figure 1). In addition, we showed that *PHIP* is overexpressed in both triple-negative and basal-like breast cancer using TCGA cohort analysis.

A major outstanding issue concerns whether the PHIP protein is druggable. PHIP does not have enzymatic activity that can be readily targeted. The PHIP protein does contain two bromodomain motifs, one of which was recently shown to be targetable [9]. However, whether PHIP's bromodomains are functional had not been conclusively demonstrated. Our studies showed that PHIP co-localizes and physically interacts with H4K91ac, an activating histone modification [2]. These results are complemented by studies in Dr. Aladjem's laboratory showing important roles for PHIP in regulating DNA replication [10].

Taken together, these studies have identified an exciting role for PHIP in the progression of melanoma and other cancers, and as a potential therapeutic target. In the biomarker realm, PHIP is one of few markers whose prognostic role has been demonstrated at both the DNA and protein levels. The enrichment of PHIP in melanoma metastases is quite unprecedented, and suggests the important survival advantage it confers to melanoma cells, resulting in the progressive copy number elevations observed in melanoma progression. Separately, the enrichment of PHIP in various molecular subtypes identifies it as a rational target for therapy of tumors in which therapeutic targeting has been difficult to achieve (e.g., *NRAS*-mutant melanoma and driver-negative tumors). In summary, PHIP is a highly versatile protein with diverse cellular functions, supporting its enrichment in distinct cancer subtypes, and representing a novel target for therapy. Additional research will be required to fulfill the promise of PHIP both as a biomarker and as a target for cancer therapy.
